# H3Africa comes of age

**Published:** 2015

**Authors:** George A Mensah, Emmanuel K Peprah, Uchechukwu KA Sampson, Richard S Cooper

**Affiliations:** Center for Translation Research and Implementation Science (CTRIS), National Heart, Lung, and Blood Institute, National Institutes of Health, Bethesda, Maryland, USA; Center for Translation Research and Implementation Science (CTRIS), National Heart, Lung, and Blood Institute, National Institutes of Health, Bethesda, Maryland, USA; Center for Translation Research and Implementation Science (CTRIS), National Heart, Lung, and Blood Institute, National Institutes of Health, Bethesda, Maryland, USA; Department of Public Health Sciences, Stritch School of Medicine, Loyola University, Chicago, Maywood, Illinois, USA

With the advent of technology that made possible large-scale sequencing and genotyping studies, it quickly became apparent that the demographic history of our species had been recorded in the genome and we could reconstruct our wanderings across the globe by studying DNA. While the vast majority of genetic variation is shared among continental populations, the most prominent finding from these early surveys of regional populations was the substantially greater degree of heterozygosity found in contemporary African populations.^1^ The ‘out of Africa’ story has long been a central dogma in paleoanthropology, and the rich cultural and linguistic heritage of the continent has also been well documented; yet the implications of this phase of human history for biomedicine had never been fully appreciated.

Major research endeavours, including the HapMap Project,^2^ the 1,000 Genomes,^3^ and more recently the African Genome Diversity Project,^4^ have exhaustively demonstrated that African populations exhibit greater sequence variation, shorter population-shared haplotype lengths, and less linkage disequilibrium than other continental groups, all a result of the long history of human occupation of sub-Saharan Africa (SSA).^2^-^4^ At the same time, the biomedical research community was confronted with the glaring reality that genetic research on both normal phenotypic traits as well as conditions of medical interest had been grossly neglected in Africa. As a result, in 2010, the US National Institutes of Health and the UK Wellcome Trust joined forces to launch an unprecedented research programme, now known as Human Heredity and Health in Africa, or H3Africa.^5^

H3Africa has established an ambitious agenda that includes not only large-scale, disease-specific research projects but training and capacity development as well. In this special issue of *Cardiovascular Journal of Africa*, some of the initial efforts of the disease-oriented programme of H3Africa are described.

The articles document the enormous effort contributed by staff at the NIH and the Wellcome Trust and teams of scientists in Africa and their collaborators in Europe and North America to bring state-of-the art genomic science to Africa. These reports primarily reflect presentations that were made at the 30 May 2014 workshop of the H3Africa cardiovascular disease (CVD) working group held in conjunction with the fourth H3Africa consortium meeting in Cape Town, South Africa.^6^

The primary workshop objectives were to review the burden of CVD in sub-Saharan Africa, advance our understanding of the genetic underpinnings of the major CVDs in Africa, and strengthen collaborations among the H3Africa research teams and other researchers using novel genomic and epidemiological tools in the assessment of CVD in Africa.^6^ As is readily apparent, CVD research consortia sponsored by H3Africa are well on their way to becoming mature international collaborations that are capable of coordinating recruitment of participants, phenotype characterisation and analysis of genetic variation and other key physiological traits.

## Defining the historical and biomedical contest for H3Africa

H3Africa has taken on a series of daunting challenges. As confirmed above, however, the record of accomplishment on display in the collection of reports in this issue demonstrate that progress is being made – both to create a coherent intellectual framework for the research that has been initiated and to build a structure that can carry out the complex logistical and laboratory tasks that the work scope demands. However H3Africa has set an even higher standard against which it will ultimately be judged. While dedicated to capacity building and the application of the powerful new tools of genomics on the continent, as described in the White Paper,^7^ written at the launch of H3Africa, there is also a clear recognition that in a region of the world with limited resources for biomedical research and healthcare delivery, all scientific ventures in SSA must take account of this context of under-development. This perspective was clearly articulated in the final paragraph of the H3Africa White Paper^7^

Genomics is under increasing pressure to demonstrate the value of the enormous investment… that have been made (and must) accept a direct comparison to epidemiology and public health as weapons to improve human health… Nowhere will this competition be more difficult than in Africa.^7^

For research in CVD, the bar in terms of the competition for relative efficacy is raised even higher than for many other diseases. In the last half-century, epidemiology has provided a near-complete description of the environmental factors that cause common CVD, and both preventative and therapeutic measures with great efficacy have been identified.

Wide implementation of these prevention and treatment strategies in most industrialised countries has led to dramatic declines in both coronary heart disease and stroke mortality of 60–80% in the last 50 years.^8^,^9^ At least half of the decline in CVD mortality has been accomplished by reducing population levels of risk factors.^8^,^9^ A similarly dramatic decline in CVD mortality resulting from population-level reductions of risk factors cannot be demonstrated for sub-Saharan Africa for a variety of reasons.

First, reliable survey data on CVD risk factors and incidence and mortality are virtually non-existent in sub-Saharan Africa (SSA), with the exception of the Republic of South Africa, Mauritius and the Seychelles.^10^ A recent meta-analysis of sample surveys however documented the substantial burden of hypertension, and the all-too-obvious low rates of treatment and control.^11^ Likewise, as documented in three articles in this issue, stroke imposes an enormous burden on the population of SSA. Therefore, while the attempt is being made to drive biomedical research forward into wholly unchartered territory through the use of genomic technology, we must not lose sight of the historical context of the evolution of CVD and the requirement in the short run to use what we already know will spare patients premature mortality, morbidity and misery.

For reasons too obvious to require elaboration, the infrastructure of public health surveillance for CVD risk factors and primary care systems that can provide long-term treatment are missing in most of SSA. Establishing these essential components of a healthcare system must therefore rank as the most urgent priority.

How then can we situate the research agenda for genomics and the H3 programme within the real-world context of SSA? As noted above, this question weighed heavily on the minds of the scientists and staff who designed H3Africa, and the White Paper and other related documents provide insightful responses to this question. Science is a broad social movement and consistent improvement in the health and economic opportunity of any country requires a vibrant scientific community.

H3Africa has clearly injected tremendous enthusiasm into the biomedical community in Africa and has led to rapid intellectual and technical advances in many areas of biomedicine. As editorialists, we respond to the question ‘what role for genomics?’ not with skepticism but rather a plea for an appreciation of the full breadth of scientific achievement now available to those who work to reduce the burden of CVD, and a reminder that we must never forget that curiosity and skepticism are co-equal ingredients of the research process.

Progress into the translational sphere is already being made and genomics has led to multiple breakthroughs in CVD research in recent years. The discovery of genetic variants of the gene coding for PCSK9 has led directly to a major new treatment modality for hyperlipidaemia.^12^ Notably, the mutation underlying this discovery was found in an Afro-origin population, where it occurs at a frequency of 1–2%, and underscores the importance of genetic research in the highly diverse societies of SSA.

Identification of a variant in APOL1 has greatly improved our understanding of the excess rates of renal failure in Afroorigin populations,^13^-^15^ again based on a mutation occurring primarily in Africans. Genomic research is also generating mounting evidence that levels of high-density lipoprotein (HDL) cholesterol are not causally related to risk of CVD.^16^ If fully verified, a demonstration of a non-causal role for HDL cholesterol would have a major impact on the approach of physicians to the treatment of serum lipid levels and re-direct an enormous research effort away from drugs intended to raise levels of this lipoprotein.

We are on the threshold of the discoveries of the molecular mechanisms underlying CVD that can improve the life of patients everywhere, and, although only on the threshold, we can confidently say that the door has been unlocked. For instance, what if in this process we were able to realise a stroke-free generation of individuals living with sickle cell disease?17 What if we were able to identify genetic or bio-molecular targets that help transform the outcomes of CVD? Compelling research questions such as these must be part of the strategic visioning in biomedical research over the next decade.^17^

Many opportunities for important discoveries of the mechanism of disease in CVD will surely be found in Africa. In the interim, however, we must endeavour to also implement evidence-based interventions that are feasible, affordable and appropriate to implement within the constraints of the SSA setting for the prevention and control of cardiovascular diseases in Africa. A priority among these interventions is those that address hypertension, diabetes, unhealthy diet, physical inactivity and tobacco use. Additionally, investments to improve the systematic collection, analysis and reporting of survey data on CVD incidence, prevalence, magnitude and trends in risk exposure, disease burden and mortality will be necessary.

The current myriad of clinical and public health challenges cannot await the promises of the genomic revolution. Active dissemination and implementation of effective interventions for prevention, treatment and control of CVD and other non-communicable diseases must be prioritised. Herein, the words of Dr Martin Luther King ring loud and true because indeed ‘… we are confronted with the fierce urgency of now... there is such a thing as being too late… this is a time for vigorous and positive action.’
